# Altered rhizosphere microbiome composition associated with B-subgenome cultivars of diploid and triploid banana plants

**DOI:** 10.1093/ismejo/wraf190

**Published:** 2025-09-15

**Authors:** Daniella Gat, Sofia Maite Arellano, Navot Galpaz, Elisa Korenblum

**Affiliations:** Institute of Plant Sciences, Agricultural Research Organization, Volcani Center, Rishon LeZion 7505101, Israel; Institute of Plant Sciences, Agricultural Research Organization, Volcani Center, Rishon LeZion 7505101, Israel; Northern Research and Development, MIGAL Galilee Research Institute, Kiryat Shmona 1101602, Israel; Institute of Plant Sciences, Agricultural Research Organization, Volcani Center, Rishon LeZion 7505101, Israel

**Keywords:** rhizosphere microbiome, host effect, *Musa* subgenomes

## Abstract

Plant genetic variation affects root phenotype and exudate composition, making it a pivotal factor in host-specific rhizosphere effects. Here we compare the rhizosphere microbiome of banana (*Musa* spp.) diploid cultivars (AA and BB), triploid cultivars derived from genome hybridizations into autotriploid cultivars (AAA), and various allotriploid cultivars (AAB and ABB) grown under field conditions to assess the influence of genome and subgenome type on the rhizosphere microbial community. Our study revealed that rhizosphere microbiomes of banana plants are significantly affected by banana genome type, presence/absence of the B-subgenome, and cultivar. Moreover, host selection strength in the assembly of the rhizosphere microbiome (i.e. rhizosphere effect) of B-subgenome-bearing banana was significantly greater than that of A-subgenome cultivars, and their rhizosphere microbial networks differed in hub membership, clustering, and node centrality measures. Thus, banana plants assemble different microbiomes in the rhizosphere according to their subgenome type. These results lay the groundwork for linking plant functional genomics and rhizosphere microbiome assembly.

## Introduction

Plant whole-genome duplication (polyploidy) is commonly linked to adaptive traits associated with the rise of novel morphological and physiological characteristics [1]. Whereas the plant microbiome is a host genotype-associated trait [2–4], evidence of plant genome duplication affecting microbiome composition is ambiguous. Ploidy has been associated with microbiome changes in wheat, strawberry, and duckweed, but not *Arabidopsis* [5–8]. If ploidy does affect the plant microbiome, we hypothesize that plants harbouring different subgenome types will be associated with distinct rhizosphere microbiomes. The domestication process of bananas (*Musa* spp.) involved rounds of independent whole-genome duplication, with both intra- and interspecific hybridizations between *Musa acuminata* (AA genome) and *Musa balbisiana* (BB genome); this generated the diversification seen today, where most of the varieties are autotriploids (AAA genomes) and allotriploids (AAB and ABB genomes) [9, 10]. In banana roots, gene expression differences between allo- and autotriploid cultivars are largely driven by the presence or absence of the B subgenome; for instance genes with expression dominance in the B-subgenome are involved in ethylene biosynthesis and the biosynthesis of secondary metabolite pathways [11, 12]. These secondary metabolites may function as semiochemicals, modulating the rhizosphere effect in various plants [13]. We therefore evaluated whether and how plant genome structure and variation (ploidy, B-subgenome content, genome type, and cultivar) affect the bacterial and fungal community structures in the rhizosphere of 14 banana cultivars with five different genome types (AA, BB, AAA, AAB, and ABB), all grown in the same soil type in a banana collection plantation for 6 years (see Supplementary information for experimental details; Tables S1 and S2, and Figs S1 and S2). We divided the banana plants into two groups: A-subgenome group for plants containing subgenome A only (AA, AAA), and B-subgenome group for plants containing a set of subgenome B (AAB, ABB, BB).

**Figure 1:**
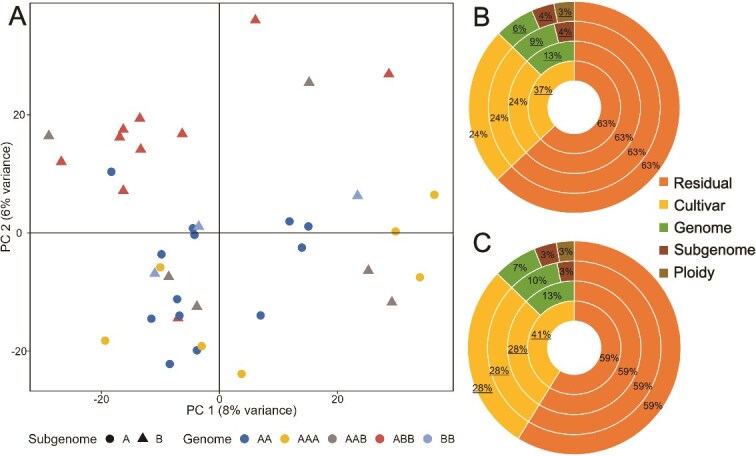
Effect of banana genome variation on banana rhizosphere microbiome composition. (A) PCA of banana plant rhizosphere bacterial community composition, with rhizosphere samples of the B-subgenome group differing from the A-subgenome group along PC2. PERMANOVA of the different banana genome parameters (ploidy, genome type, subgenome type, and cultivar) affecting host bacterial (B) and fungal (C) community variance in the rhizosphere; percentages indicate the *R*^2^ values of variance explained by each parameter; underline type indicates significance at *P* value <0.05.

Banana plants are giant perennial herbs with an underground corm (rhizome) that produces suckers (new daughter plants from the same banana plant) and cord roots (adventitious main roots) [14]. Under field conditions, the rhizomes’ location changes on a seasonal basis according to sucker selection. The rhizosphere microbiome is primarily shaped by soil characteristics [15]. At the outset of our analysis, we evaluated the rhizosphere effect by comparing the rhizosphere microbiome with the bulk soil microbiome for each banana plant. We confirmed that the soil and rhizosphere bacterial communities differed significantly in their community composition (PERMANOVA, *P* value = 0.001). These differences are also evident in the principal component analysis (PCA) plot (Fig. S3A), but we observed no significant difference in their alpha diversities (Fig. S3B). Bacterial alpha diversity is generally depleted in the rhizosphere compared to bulk soil in agricultural lands planted with vegetable crops; in contrast, in grasslands, forest ecosystems, and mineral-fertilized soils, the microbial diversity of bulk soil and rhizosphere was found similar [15]. PCA of the rhizosphere microbiome showed that banana subgenome type affects the rhizosphere bacterial community structure, with the B-subgenome plants separating from the A-subgenome ones along PC2, and the rhizosphere microbiome of ABB cultivars showing the most distinct clustering (Fig. 1A). We then examined the effect of different plant host parameters (cultivar, subgenome, genome, and ploidy level) on the bulk soil and rhizosphere microbiome compositions using Permutation Analysis of Variance (PERMANOVA). None of these parameters, or plant location in the plot (Table S3), significantly affected bulk soil microbiome composition; however, genome structure variation (ploidy, subgenome type, and genome) consistently cumulatively affected the rhizosphere’s bacterial community composition (Fig. 1B, Table S3). Whereas ploidy significantly affected the rhizosphere microbiome of banana (Fig. 1B, outermost ring), *Arabidopsis* genotypes with different ploidy levels have the same root microbiome [5]. The total genetic variation across the banana plant cultivars explained 37% of the rhizosphere bacterial community variance in a cumulative manner (*R*^2^ = 0.37, *P* value = 0.003, innermost ring, Fig. 1B). However, combined variations for genome type, subgenome, and ploidy explained a total of 13% of the variance (6% genome type, 4% subgenome type, and 3% ploidy; *P* value <0.05), with the remaining 24% for cultivar being reduced to marginal insignificance (*P* value = 0.066). Fungal rhizosphere communities were also significantly affected by total plant genetic variation (*R*^2^ = 0.41, *P* value = 0.014), yet accounting for genome type, subgenome, and ploidy did not reveal any such significant effect; cultivar remained the significant parameter affecting fungal community composition (*R*^2^ = 0.28, *P* value = 0.025, Fig. 1C).

The core microbiome of banana plants, generally defined as bacteria and fungi that frequent most banana plants worldwide, was suggested to provide the basis for microbiome management as a means to prevent diseases [16, 17]. Comparing the microbiome composition of the banana rhizosphere to previously described banana core microbiome data sets, we found 15 bacterial taxa and 1 fungal taxon present in our dataset out of 16 and 7 bacterial and fungal taxa, respectively, previously described as core members of the banana rhizosphere microbiome [16, 17]. This result further affirms the conclusions of these previous studies regarding the universality of these taxa in banana root microbiome. However, despite identical core microbial membership between A- and B-subgenome plants in our dataset, the cumulative relative abundance of core taxa was significantly higher in the former (Fig. S4).

Using cultivar as an inclusive term for all genetic variations, we identified 1223 bacterial amplicon sequence variants (ASVs) associated with the 14 banana cultivars (linear mixed model MaAsLin2, adj. *P* value <0.05, Table S4). When sorting the cultivars by subgenome type, the cultivar-associated ASVs were not evenly distributed between the two subgenomes: B-subgenome cultivars were characterized by a higher frequency of differentially abundant *Alphaprotebacteria* and *Gammaproteobacteria*, whereas A-subgenome cultivars had a higher frequency of *Polyangia* and *Gemmatimonadetes* (Fig. S5, Table S4). A similar observation was found for the rhizosphere fungi, with a total of 713 ASVs significantly associated with at least one banana cultivar, among which were potential plant pathogens, such as: *Botryosphaeria, Setophoma, Curvularia americana,* and *Rhizoctonia solani*. Other taxa were putative plant growth promoting fungi (e.g. *Aspergillus fumigatus, Penicillium brevicompactum,* and *Mortierella).* We observed that *Agariomycetes* were significantly associated with all cultivars but two (Pissng-Awak and Aacv-Rose), *Dothideomycetes*, to which some of the possible plant pathogens belong, were uniquely more abundant in B-subgenome plants while *Sordariomycetes* and *Eurotiomycetes*, to which many of the potentially growth promoting taxa belong, were more frequently found to be abundant in A-subgenome plants (Fig. S5  Table S4).

Comparative network analysis between rhizosphere microbiomes, including bacteria and fungi, of A- and B-subgenome plants revealed a significantly different membership of hub taxa and different degrees of centrality of the ASVs in each network (Jaccard <<1), as well as an adjusted Rand index that differed significantly from 0, suggesting different clustering for each plant subgenome-type network (Fig. S6A, Table S5). Comparing the ASVs forming network hubs, there were some taxonomic differences between A- and B-subgenome plants: the B-subgenome hubs were more varied and included unique taxa belonging to the phyla *Spirochaetota*, *Planctomycetota*, *Patescibacteria*, *Nitrospirota*, and *Methylomirabilota*; A-subgenome hubs included *Glomeromycota* and *Fibrobacterota* (Fig. S6B).

**Figure 2:**
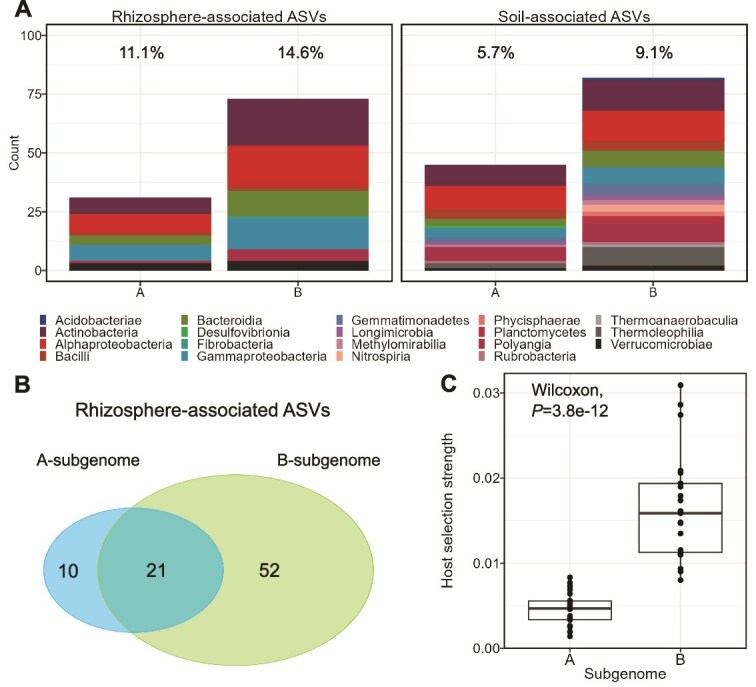
Rhizosphere effect of B-subgenome plants is stronger than that of A-subgenome plants. (A) ASVs significantly associated with either the rhizosphere or soil around banana roots by taxonomy using MaAsLin2 (adj. *P* < 0.05). Y-axis shows the number of ASVs; percentage over each bar indicates their total relative abundance in the data set, per subgenome. (B) Venn diagram showing the number of shared and unique rhizosphere-associated ASVs in A- and B-subgenome plants. (C) Calculated host selection strength in A- and B-subgenome plants based on differentially abundant ASVs in plants of each subgenome group.

To estimate the host selection strength [18] of each subgenome in the assembly of the rhizosphere microbiome, i.e., the rhizosphere effect of B-subgenome vs. A-subgenome plants, we first determined bacterial ASVs that associate significantly with the rhizosphere compared to the bulk soil, for each subgenome, using MaAsLin2 (adj. *P* value <0.05, Table S6). More ASVs were significantly associated (enriched or depleted) with B-subgenome-bearing plants (73 and 82, respectively) compared to plants containing only the A-subgenome (31 and 45, respectively) (Fig. 2A). A total of 21 ASVs were positively associated with the rhizosphere of both A- and B-subgenome plants, but the B-subgenome rhizosphere was enriched with 52 unique ASVs, more than five times the number of unique enriched ASVs in the rhizosphere of A-subgenome plants (Fig. 2B). Based on these results, we calculated host selection strength per sample and observed a significant difference (Wilcoxon, *P* value <0.001) between A and B-subgenome plants, with mean values of 0.004 ± 0.002 and 0.014 ± 0.005, respectively (Fig. 2C). Fungal sequencing depth was limited in some soil and a few rhizosphere samples (Fig. S2), thus further data are needed to clarify specifically host selection strength for fungal members.

B-subgenome plants show increased expression of genes associated with secondary metabolite biosynthesis in the root tips compared to A-subgenome plants [12]. Despite the evidence for functional divergence between the A- and B-subgenomes at the transcriptional level in different banana plant tissues [11], previous studies comparing the microbiome of several banana cultivars did not find a genotype effect on the rhizosphere microbial composition of banana plants [16, 17, 19]. Here we observed that banana cultivars have different rhizosphere bacterial and fungal community compositions and the network analysis indicated that plants bearing B- subgenome show distinct rhizosphere bacterial and fungal interactions as compared to A-subgenome group. Moreover, we identified a stronger rhizosphere effect (host selection strength) of B-subgenome plants compared to A-subgenome plants growing in an agricultural field setting. These unique rhizospheric bacterial populations may contain differential adaptive traits that promote plant growth and protection. Functional enrichment for antimicrobial-biosynthesis genes was previously observed in *M. balbisiana* (BB genotype) root endophytic metagenome as compared to *M. acuminata* (AAA) [19]. In future work, it would be interesting to test whether the stronger rhizosphere effect found in B-subgenome plants is due to increased investment in root exudation of metabolites to shape their rhizosphere microbiome, and whether these enriched bacterial populations benefit plant defence and growth. It is predicted that climate change will decrease banana production worldwide [20]. This emphasizes the importance of understanding the mechanisms governing the rhizosphere microbiome assembly process and its effect on banana plants, for the potential improvement of new and regenerative banana-cultivation practices.

## Supplementary Material

supplementary_information_Gat_etal_2025_wraf190

Table_S2_wraf190(1)

Table_S4_wraf190

Table_S5_wraf190

Table_S6_wraf190

## Data Availability

Sequences are available on NCBI SRA database under the accession number PRJNA1229108. The code for reproducing the figures and statistical analyses can be found on GitHub (https://github.com/EK-labRhizo/Banana-genome).
