# Supplementary material for: Altered rhizosphere microbiome composition associated with B-subgenome cultivars of diploid and triploid banana plants
Source: ISME J. 2025 Sep 15;19(1):wraf190. doi: 10.1093/ismejo/wraf190 (PMC12448422; doi:10.1093/ismejo/wraf190)
Supplement: supplementary_information_Gat_etal_2025_wraf190 [file supplementary_information_gat_etal_2025_wraf190.docx]

Supplementary Information

**Altered rhizosphere microbiome composition associated with B-subgenome cultivars of diploid and triploid banana plants**

**Daniella Gat^1^, Sofia Maite Arellano^1^, Navot Galpaz^2^, Elisa Korenblum^1,^***

^1^ Institute of Plant Sciences, Agricultural Research Organization, Volcani Center, Rishon LeZion, Israel

^2^ Northern Research and Development, MIGAL Galilee Research Institute, Kiryat Shmona, Israel

*Corresponding author: Elisa Korenblum, Institute of Plant Sciences, Agricultural Research Organization, Volcani Center, 68 HaMaccabim Road, POB 15159 Rishon LeZion 7505101, Israel. Email: elisak@volcani.agri.gov.il

The supplementary information includes Methods, Figures S1-S6 and Tables S1-S6

**Methods**

Rhizosphere and soil sampling

Sampling was conducted at the Zemach research farm, situated in the Jordan Valley (32°42'15.3"N, 35°34'46.9"E), Israel. The banana collection plantation was established in 2015 (Fig. S1) and is comprised of banana cultivars gathered from various countries, all grown in a single soil type; from each cultivar, 4 banana plants were initially transferred to the plot as vegetative suckers (daughter plants) and the banana cultivars were propagated by selecting new suckers from the original banana plant every year. The field collection plot was maintained under commercial conditions for 6 years prior to root and soil sampling in this current study, which took place in 2021. Root samples from 14 cultivars (Table S1) were collected from three to four different banana plants per cultivar. In an attempt to collect active roots that represent a similar stage of plant development, we collected roots from new, 1-m tall suckers, the root portion closest to the corm base. The samples were clipped using disinfected clippers (cleaned with 70% ethanol between samples) and inserted into sterile, DNAse/RNAse free 15ml tubes. Soil samples were collected nearby (15-20 cm away) the collected roots by scooping into sterile, DNAse/RNAse free 15ml tubes. All tubes were kept in liquid nitrogen until transfer to a -80^o^C freezer.

**Table S1.** List of banana cultivars, genomes, ploidy, and presence/absence of B-type genome (subgenome).

| **Cultivar** | **Genome** | **Ploidy** | **Subgenome** |
| --- | --- | --- | --- |
| Aacv-Rose | AA | 2 | A |
| Apple-banana | AAB | 3 | B |
| Balbisiana | BB | 2 | B |
| Blue-banana | ABB | 3 | B |
| Bluggoe | ABB | 3 | B |
| Calcutta 4 | AA | 2 | A |
| Grand Naine 5/37 | AAA | 3 | A |
| Hom Thong Mokho | AAA | 3 | A |
| Lakatan | AA | 2 | A |
| M. malaccensis | AA | 2 | A |
| Natan | AAA | 3 | A |
| Pissang-Awak | ABB | 3 | B |
| Plantain | AAB | 3 | B |
| Prata Ana | AAB | 3 | B |

Rhizosphere DNA extraction

Two pieces of root, each about 2 cm long, from each plant were inserted into a Lysing Matrix E bead tube (MP Biomedicals, USA) with 500 µl of a phenol:chloroform:isoamyl mixture (25:24:1, v/v) and 500 µl CTAB buffer. The tubes were vortexed for 3 min at maximum speed using a horizontal adapter, then centrifuged at 17,000 rcf for 10 min. The aqueous phase in each tube was transferred to a new tube containing 500 µl of chloroform:isoamyl (24:1) and kept on ice; 500 µl of CTAB buffer was again added to each bead tube and the bead-beating process was repeated with the same sample. The aqueous phase was transferred to a new tube containing the chloroform:isoamyl mixture. The tubes were mixed briefly and centrifuged at 17,000 rcf for 10 min. The aqueous phase of each tube was transferred to a new tube containing PEG and glycogen; the contents were mixed and left at 4^o^C overnight. The next day, the tubes were centrifuged for 1.5 h at 17,000 rcf, the supernatant was discarded, and the pellet was suspended in 80% ethanol and re-centrifuged for 45 min at 17,000 rcf. The supernatant was discarded, the tubes were left open at 65^o^C for 2 min to remove all ethanol, and the pellet was then suspended in Tris-EDTA buffer.

Soil DNA extraction

Soil DNA was extracted by the same protocol as the rhizosphere DNA extraction, with the following minor differences: about 0.3 g of soil from the root zone of each sampled plant was used. The bead-beating step was extended to 10 min and was repeated three times for each sample. The first centrifugation step was shortened to 2 min.

PCR and barcoding

PCR amplification of the 16S rRNA gene (a proxy for bacterial taxonomy) and of the internally transcribed spacer (ITS, a proxy for fungal taxonomy) was conducted using the primer set 515F-806R for 16S and ITS1F-ITS2R for ITS [1,2]. Each forward primer was barcoded with a unique 5-mer sequence to allow pooling of samples prior to library preparation. The reaction was conducted using MyTaq HS mix (Meridian Bioscience, USA) as follows: 5 min at 95^o^C, followed by 28 cycles of 30 s at 95^o^C, 30 s at 50^o^C/52^o^C (for 16S/ITS, respectively), and 15 s at 72^o^C; this was followed by a final elongation step of 5 min at 72^o^C. All PCR products were run on an agarose gel (1.2%, w/v) to examine the amplicons for size and amount. PCR products were pooled and cleaned using Wizard SV gel and PCR clean-up system (Promega, USA). DNA concentrations were measured using Qubit (Thermo Fisher Scientific, USA).

DNA amplicon sequencing

DNA quality was assessed using TapeStation (Agilent, USA). Two different libraries were constructed: one for ITS and one for 16S amplicons. Following adapter ligation, the libraries were amplified for an additional 14 cycles and sequenced using a MiSeq System (Illumina, USA, V2, 500 cycles, paired-end) at the Crown Institute for Genomics, G-INCPM facility at the Weizmann Institute of Science, Rehovot, Israel.

Sequence pre-processing

The sequences were demultiplexed using cutadapt v.4.5 and processed using DADA2 (v.1.26, [3]) according to the suggested pipeline. However, merging forward and reverse reads resulted in too few consensus reads (ca. 52%); therefore, we continued the analysis using only the reverse reads (Table S2). Taxonomy assignment was conducted using the SILVA nr99 train set (v. 138.0) and UNITE (v. 9) for 16S rRNA genes and ITS, respectively. Mitochondria and plastid sequences, as well as unclassified phyla, were manually removed. In addition, amplicon sequence variants (ASV) of the lowest 5-percentile read counts abundance and with a prevalence lower than 5% of the samples (i.e., present in less than 4 rhizosphere samples or 4 soil samples) were removed to decrease sparsity. Rhizosphere samples with a total read count under 3000 and 1000 for bacteria and fungi, respectively, were discarded, in addition a single sample was identified as contaminated and was discarded (R22, Prata Ana, see Table S2). Similarly, soil samples with total read counts under 2000 and 500 for bacteria and fungi, respectively, were removed. Ultimately, rhizosphere count tables included 5426 bacterial ASV in 40 samples and 845 fungal ASV in 37 samples. Soil count tables included 4505 bacterial ASV in 40 samples and 666 fungal ASV in 29 samples. Based on rarefaction curves (Fig. S2), ITS sampling depth in soils was insufficient to capture the full fungal diversity in some samples. We therefore decided to conduct multivariable associations between rhizosphere and soil only on bacteria.

Statistical analysis

Statistical analysis was conducted using R and mothur [4]. To account for uneven sequencing depth, the bacterial and fungal ASV counts were rarefied to the size of the smallest sample for soil and rhizosphere. Rarefaction was repeated randomly for 1000 iterations, at the end of which a mean count was calculated for each ASV in each sample [5]. Transformation of the rarefied counts was conducted using centred log-ratio (clr) which followed zero-correction using geometric Bayesian multiplicative transformation (zCompositionsv.1.4.1 package), to account for the compositionality of the data [6]. A PERMANOVA test based on Euclidean distances was conducted on the transformed data, separated into rhizosphere and soil samples and into fungi and bacteria, looking for batch effects due to different DNA-extraction dates, soil microbiome homogeneity (location in the plot), and position on the PCR plate. Only extraction date was found to significantly affect the rhizosphere and soil microbiomes, and we therefore used MaAsLin [7] to find all bacterial ASV that were significantly associated with extraction date (adj. *P* value < 0.2) and removed them from the raw counts table. No such action was taken for fungal ASV due to their initial scarcity; instead, we used the "strata" argument for PERMANOVA of the rhizosphere fungal community. Rarefaction and transformation were then repeated as described above, and PERMANOVA on the filtered data set showed no significant batch effect (Table S3). All presented bacterial community composition analyses were conducted on the filtered rhizosphere and soil ASV count tables. Principal component analysis (PCA) ordination relied on Euclidean distances [8]. For cultivar-associated ASV, we repeated the MaAsLin analysis on the bacterial and fungal rhizosphere datasets, each time defining a different cultivar as the reference set, extraction date was set as a random effect for the fungal dataset.

For rhizosphere-associated bacterial ASV, we defined the plant number as a random effect, thus comparing communities between bulk soil and rhizosphere at the same location, and reducing spatial variation effects.

Core taxa comparison was conducted based on the findings of [9,10], with comparisons performed at the lowest available taxonomic level for bacteria and fungi that were defined as core taxa in the relevant plant compartments, i.e., endo- and ecto-rhizosphere. For bacteria, we used the taxa appearing in Figure 6 in [9], for a total of 16 taxa; for fungi, we used the taxa in Figure S9 in [10], for a total of 7 taxa.

To enable a comprehensive observation of the entire microbial community, ASV count tables of the two kingdoms were combined for network analysis. Network analysis was conducted for samples representing plants containing only the A-subgenome and plants with the B-subgenome on raw, un-rarefied, combined bacterial and fungal counts by selecting the top 1000 ASV with the highest variance, using NetCoMi package, and applying the spiec-easi algorithm with internal clr-transformation [11]. Hub taxa were identified using Eigen centrality, and clusters were computed using the Louvain algorithm. Alpha diversity indices were calculated using mothur by repeated subsampling of the raw read table. Plotting and general data handling were done with the packages ggplot2, ggpubr, VennDiagram, ggsci, and tidyverse [12–15].


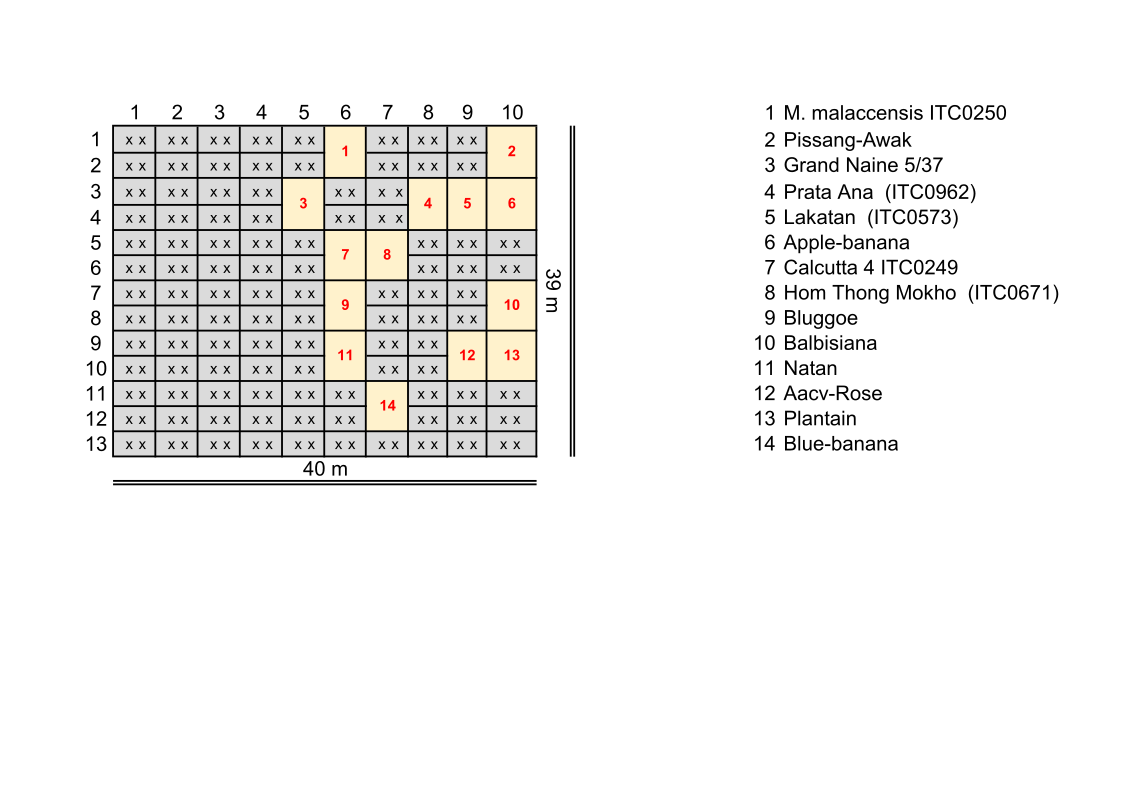


**Figure S1.** Map of the banana cultivar collection plantation. Each cell marks a single mat (cluster containing mother plants and suckers) with two mother trees per mat (x marks a tree with its suckers). The spacing between banana mats in a column is 3 m and 4 m between the columns; the 14 cultivars used in this study are numbered, 4 trees per cultivar.


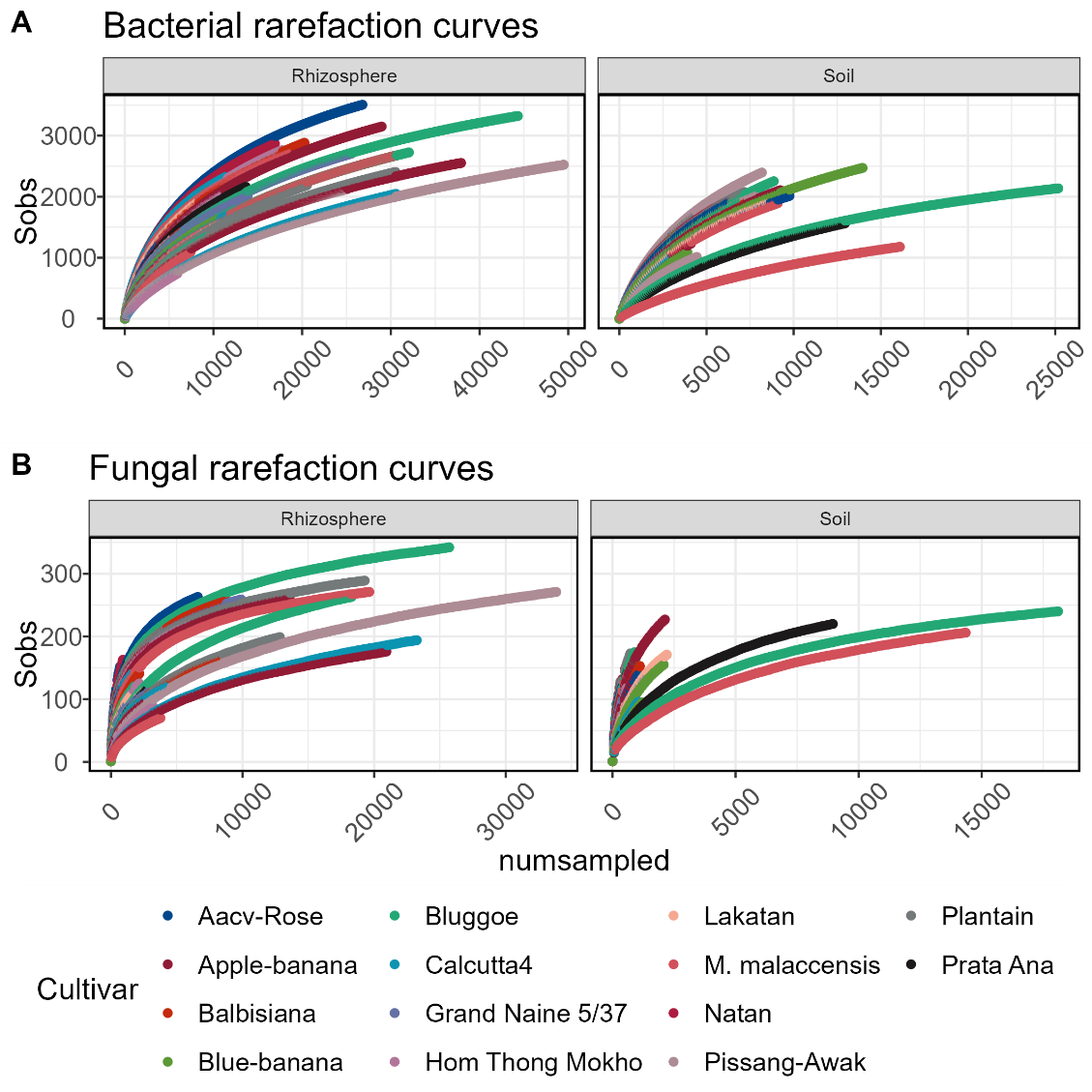


**Figure S2.** Rarefaction curves of rhizosphere and soil samples for bacterial ASV (A) and fungal ASV (B).

**Table S3.** PERMANOVA results of rarefied, filtered data sets. (A) Effect of the terms: ploidy, subgenome type, genome, and cultivar, examined together in that order. (B) Examination of batch effect by extraction date, following removal of ASV associated with this effect. (C) Examination of soil microbiome homogeneity in the plot – no effect of location on the soil microbiome.

**A**

| Parameter | Df | Sum of squares | *R*^2^ | *F* | *P* value | Data set |
| --- | --- | --- | --- | --- | --- | --- |
| Ploidy | 1 | 1159 | 0.03 | 1.1 | 0.26 | Rhizosphere fungi |
| Subgenome type | 1 | 1430 | 0.03 | 1.3 | 0.07 |  |
| Genome | 2 | 2834 | 0.07 | 1.3 | 0.16 |  |
| Cultivar | 9 | 12057 | 0.28 | 1.2 | 0.03 |  |
| Ploidy | 1 | 889 | 0.04 | 1.0 | 0.24 | Soil fungi |
| Subgenome type | 1 | 1148 | 0.05 | 1.3 | 0.02 |  |
| Genome | 2 | 1809 | 0.07 | 1.1 | 0.7 |  |
| Cultivar | 9 | 8402 | 0.33 | 1.0 | 0.275 |  |
| Ploidy | 1 | 4182 | 0.03 | 1.2 | 0.036 | Rhizosphere bacteria |
| Subgenome type | 1 | 4680 | 0.03 | 1.4 | 0.014 |  |
| Genome | 2 | 8061 | 0.06 | 1.2 | 0.024 |  |
| Cultivar | 9 | 32309 | 0.24 | 1.1 | 0.073 |  |
| Ploidy | 1 | 2125 | 0.02 | 0.9 | 0.93 | Soil bacteria |
| Subgenome type | 1 | 2861 | 0.03 | 1.2 | 0.015 |  |
| Genome | 2 | 4869 | 0.05 | 1.0 | 0.29 |  |
| Cultivar | 8 | 19344 | 0.22 | 1.0 | 0.28 |  |

**B**

| Parameter | Df | Sum of squares | *R*^2^ | *F* | *P* value | Data set |
| --- | --- | --- | --- | --- | --- | --- |
| Extraction date | 7 | 9132 | 0.21 | 1.1 | 0.07 | Rhizosphere fungi |
|  | 7 | 6801 | 0.28 | 1.1 | 0.031 | Soil fungi |
|  | 7 | 24780 | 0.19 | 1.0 | 0.32 | Rhizosphere bacteria |
|  | 7 | 17337 | 0.20 | 1.0 | 0.13 | Soil bacteria |

**C**

| Parameter | Df | Sum of squares | *R*^2^ | *F* | *P* value | Data set |
| --- | --- | --- | --- | --- | --- | --- |
| Plot column | 1 | 1031 | 0.04 | 1.2 | 0.16 | Soil fungi |
| Plot row | 1 | 891 | 0.04 | 1.0 | 0.37 |  |
| Plot column | 1 | 2424 | 0.03 | 1.0 | 0.35 | Soil bacteria |
| Plot row | 1 | 2521 | 0.03 | 1.1 | 0.22 |  |


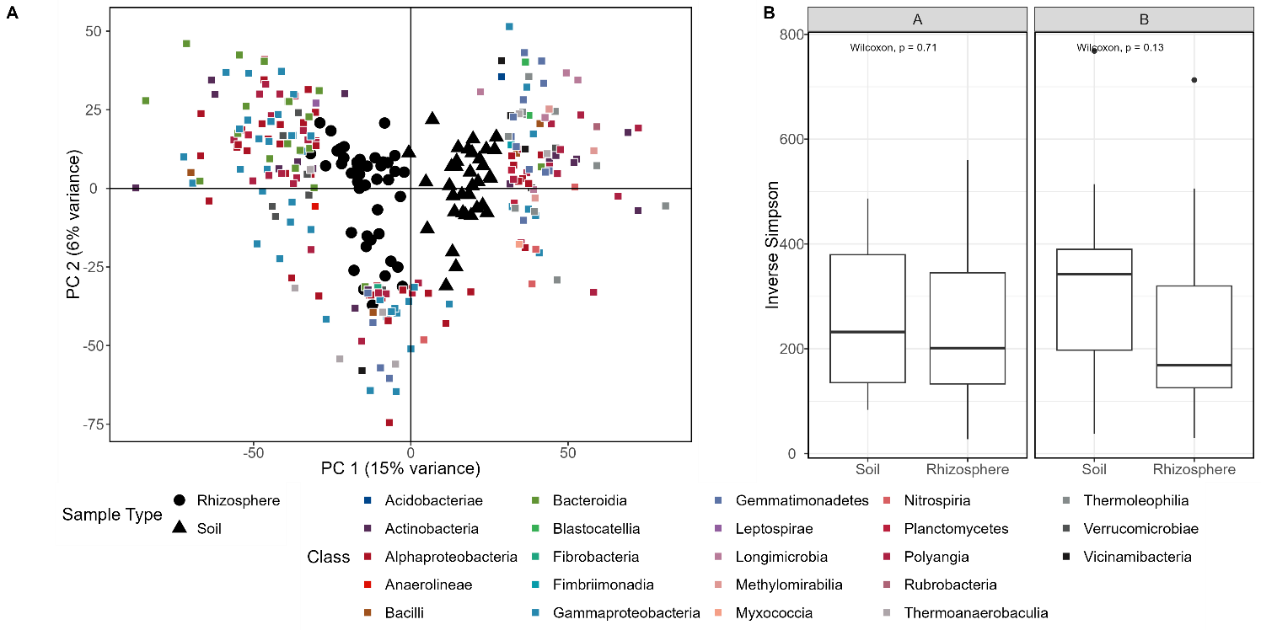


**Figure S3.** Soil and rhizosphere microbiomes of all banana mats (cluster containing mother plants and suckers) analysed in this study. (A) PCA based on bacterial community composition of banana rhizosphere and bulk soil. Loadings of ASV that are significantly associated with rhizosphere compared to soil are represented by square symbols, coloured according to class (see Table S6). (B) Inverse Simpson diversity of bacterial communities of soil and rhizosphere, displayed by plant subgenome.


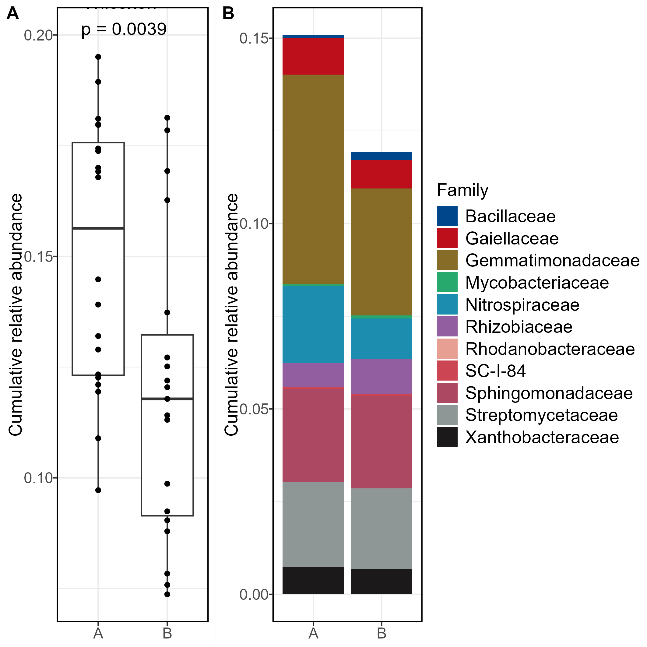


**Figure S4.** Detected core taxa of the Musa microbiome as defined by [9,10]. (A) Cumulative relative abundance of core bacterial taxa in banana plants from this study, divided by subgenome type. (B) Taxonomic composition (at the family level) of the defined core taxa presented in panel A.


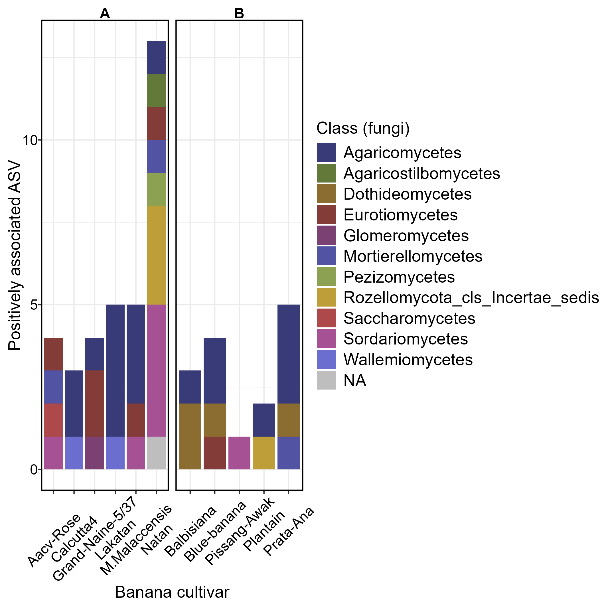

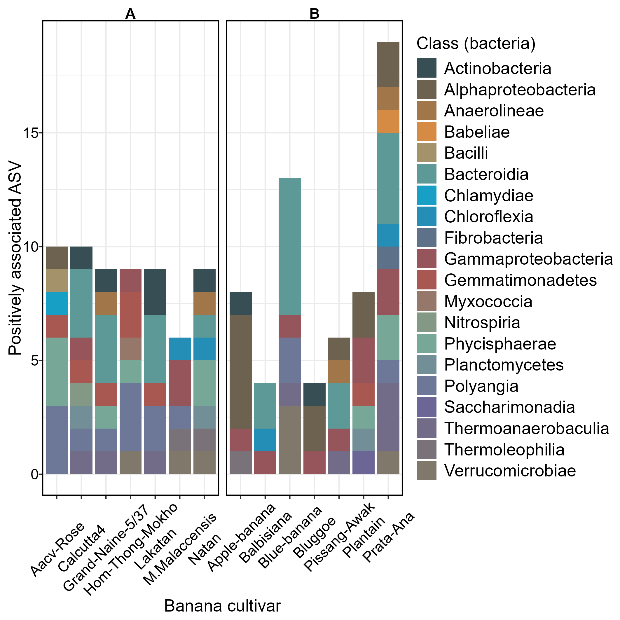


**Figure S5.** Bacterial (left) and fungal (right) ASV significantly associated with the different banana cultivars, presented at the class level and displayed by plant subgenome type (Table S4). In fungi, fewer cultivars are represented due to lower number of samples that passed the pre-processing filtration steps.


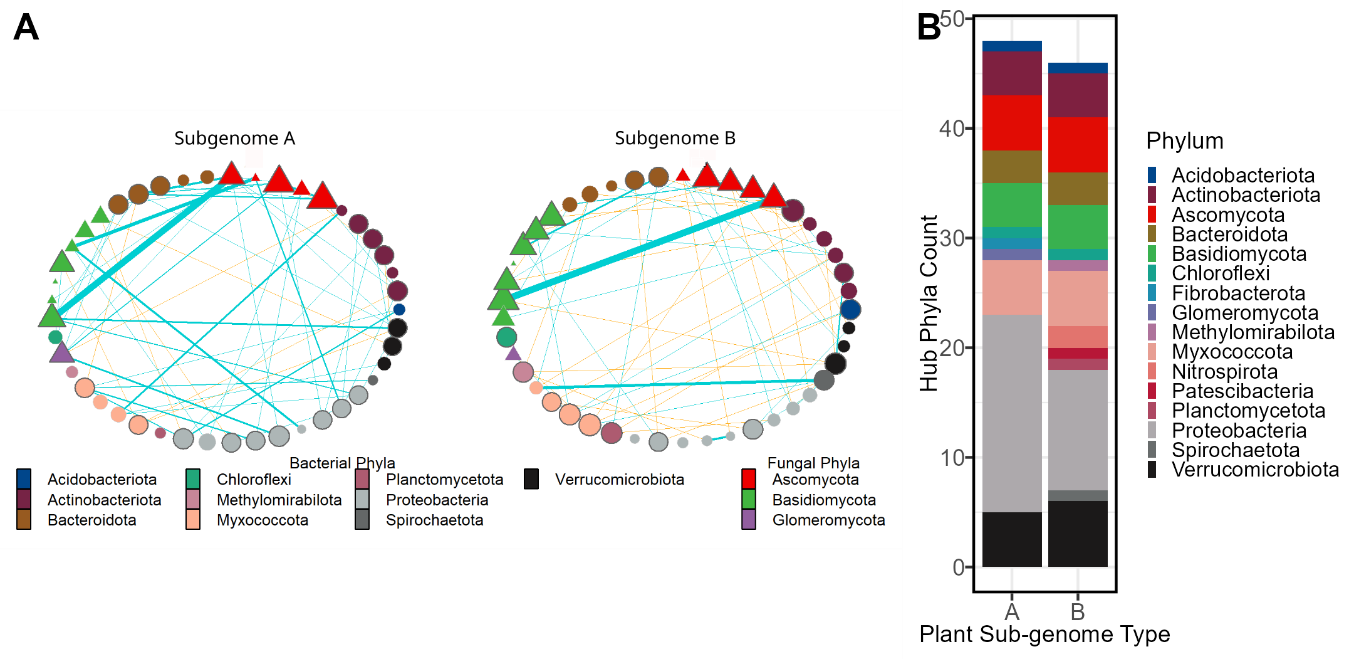


**Figure S6.** Microbial network of the 1000 most variable ASV in A- and B-subgenome plants. (A) The top 25 ASV showing the highest connectivity in each subgenome type (each network displays the union of ASV with highest connectivity from both groups: 20 ASV key to A-subgenome group, 20 ASV key to B-subgenome group and 5 ASV common to both groups, a total of 45 ASV). Node sizes correspond to the Eigen value of the nodes in each network (A- or B-subgenome). Node colour corresponds to the phylum, and edge colours correspond to association type (blue – positive, yellow – negative), edge weights indicate the relative strength of the association, thicker edges correspond to stronger association. Node shape corresponds to the taxonomic kingdom (circle – bacteria, triangle – fungi), and node outline indicates hub taxa (dark – hub, light – non-hub), 24 hubs presented in A-subgenome network and 23 hubs presented in B-subgenome network. (B) Bar chart showing all hub ASV in each network by phylum (Table S5).

**Supplementary Tables**

**Table S2.** Summary of sequence pre-processing and final total read counts per sample.

**Table S4.** Cultivar-associated bacterial and fungal ASV in rhizosphere samples.

**Table S5.** Network nodes (ASV) and their closeness, Eigen, and betweenness indices for A- and B-subgenome networks.

**Table S6.** Soil/rhizosphere-associated ASV (positive coefficient indicates enrichment in soil, negative coefficient indicates enrichment in rhizosphere).

**References**

1. Smith Dylan P. AND Peay KG. Sequence depth, not PCR replication, improves ecological inference from next generation DNA sequencing. *PLoS One* 2014;**9(2)**.

2. Caporaso JG, Lauber CL, Walters WA *et al.* Global patterns of 16S rRNA diversity at a depth of millions of sequences per sample. *PNAS* 2011;**108**:4516–22.

3. Callahan BJ, McMurdie PJ, Rosen MJ *et al.* DADA2: High-resolution sample inference from Illumina amplicon data. *Nat Methods* 2016;**13**:581–3.

4. Schloss PD, Westcott SL, Ryabin T *et al.* Introducing mothur: open-source, platform-independent, community-supported software for describing and comparing microbial communities. *Appl Environ Microbiol* 2009;**75**:7537–41.

5. Schloss PD. Rarefaction is currently the best approach to control for uneven sequencing effort in amplicon sequence analyses. *mSphere* 2024;**9**:e00354-23.

6. Gloor GB, Macklaim JM, Pawlowsky-Glahn V *et al.* Microbiome datasets are compositional: and this is not optional. *Front Microbiol* 2017;**8**:2224.

7. Mallick H, Rahnavard A, McIver LJ *et al.* Multivariable association discovery in population-scale meta-omics studies. Coelho LP (ed.). *PLoS Comput Biol* 2021;**17**:e1009442.

8. Oksanen J, Blanchet, F. Guillaume Kindt R, Legendre P *et al.* Vegan: community ecology package. R package version 1.17-4. 2007.

9. Birt HWG, Pattison AB, Skarshewski A *et al.* The core bacterial microbiome of banana (Musa spp.). *Environ Microbiome* 2022;**17**:46.

10. Birt HWG, Pattison AB, Skarshewski A *et al.* The core fungal microbiome of banana (Musa spp.). *Front Microbiol* 2023;**14**, DOI: 10.3389/fmicb.2023.1127779.

11. Peschel S, Müller CL, von Mutius E *et al.* NetCoMi: network construction and comparison for microbiome data in R. *Brief Bioinform* 2021;**22**:bbaa290.

12. Kassambara A. ggpubr: “ggplot2” Based Publication Ready Plots. 2020.

13. Wickham H. ggplot2: Elegant Graphics for Data Analysis. 2016.

14. Chen H, Boutros PC. VennDiagram: a package for the generation of highly-customizable Venn and Euler diagrams in R. *BMC Bioinformatics* 2011;**12**:35.

15. Wickham H, Averick M, Bryan J *et al.* Welcome to the Tidyverse. *J Open Source Softw* 2019;**4**:1686.
